# Hub Patterns-Based Detection of Dynamic Functional Network Metastates in Resting State: A Test-Retest Analysis

**DOI:** 10.3389/fnins.2019.00856

**Published:** 2019-09-11

**Authors:** Xin Zhao, Qiong Wu, Yuanyuan Chen, Xizi Song, Hongyan Ni, Dong Ming

**Affiliations:** ^1^Department of Biomedical Engineering, College of Precision Instruments and Optoelectronics Engineering, Tianjin University, Tianjin, China; ^2^Tianjin International Joint Research Center for Neural Engineering, Academy of Medical Engineering and Translational Medicine, Tianjin University, Tianjin, China; ^3^Department of Radiology, Tianjin First Center Hospital, Tianjin, China

**Keywords:** metastate, dynamic functional connectivity, structural network, clustering analysis, node centrality, hubs

## Abstract

The spontaneous dynamic characteristics of resting-state functional networks contain much internal brain physiological or pathological information. The metastate analysis of brain functional networks is an effective technique to quantify the essence of brain functional connectome dynamics. However, the widely used functional connectivity-based metastate analysis ignored the topological structure, which could be locally reflected by node centrality. In this study, 23 healthy young volunteers (21–26 years) were recruited and scanned twice with a 1-week interval. Based on the time sequences of node centrality, we promoted a node centrality-based clustering method to find metastates of functional connectome and conducted a test-retest experiment to assess the stability of those identified metastates using the described method. The hub regions of metastates were further compared with the structural networks’ organization to depict its potential relationship with brain structure. Results of extracted metastates showed repeatable dynamic features between repeated scans and high overlapping rate of hub regions with brain intrinsic sub-networks. These identified hub patterns from metastates further highly overlapped with the structural hub regions. These findings indicated that the proposed node centrality-based metastates detection method could reveal reliable and meaningful metastates of spontaneous dynamics and indicate the underlying nature of brain dynamics as well as the potential relationship between these dynamics and the organization of the brain connectome.

## Introduction

The functional brain connectome, considering the brain as a complex network, indicates the spatial distributions and integrated organizations. Resting-state functional magnetic resonance imaging (rs-fMRI) can provide these kinds of intrinsic information of brain function (Biswal et al., 1995; Cordes et al., 2001) through measuring the synchronization between temporal fluctuations across spatially separated brain regions, which are known as functional connectivity (FC). It is the most basic measure and has been widely used in physiology (Damoiseaux et al., 2008; Betzel et al., 2014; Chen et al., 2017, 2018) and pathology (Bing et al., 2010; Veer, 2010; Widjaja et al., 2013; Ham et al., 2015; Su et al., 2015; Zhuo et al., 2018). More importantly, the brain function in resting state also reveals dynamics or temporal distributions of brain connections, which spontaneously change from seconds to minutes (Chang and Glover, 2010; Calhoun et al., 2014), which is called dynamic FC. Dynamic FC provides a novel insight into brain function which has been proved to contain useful information (Viviano et al., 2017; Xia et al., 2019) and can even be complementary to traditional static FC (Liégeois et al., 2019). It also enables us to better understand the behavior of different subnetworks (Al-Sharoa et al., 2019) and contains the intrinsic neural activities (Hutchison et al., 2013b) associated with the brain functional (Syed et al., 2017; He et al., 2018) or structural organizations (Shen et al., 2015; Cabral et al., 2017). Though dynamic FC presents a promising way to uncover the mysterious activates in human brain function, it is still unclear how brain function dynamically changes.

Metastate in the human brain is an interesting idea with which to describe the spontaneous fluctuation of FC as well as functional networks (Allen et al., 2014; Shakil et al., 2016; Shine et al., 2016; Vidaurre et al., 2017) and originates from a typical concept, “microstates,” in electrophysiological studies (Gale, 1983; Lehmann et al., 1987). Metastates are considered as the certain brain FC patterns or brain states that repeatedly appear over and over again in the scanning period and can somehow represent those microstates at mesoscale. Increasing evidence has shown that the occurrence of transition between metastates contains meaningful information about normal aging and schizophrenia (Hansen et al., 2015; Yu et al., 2015; Shakil et al., 2016) and shows great potential regarding intrinsic interactions and complicated organizations (Hutchison et al., 2013a) of brain function (Hutchison et al., 2013a; Andrew and Michael, 2015).

Based on the sliding windowed correlations, plenty of previous studies applied whole brain FC-based clustering to represent and detect brain metastates (Allen et al., 2014; Hansen et al., 2015; Yu et al., 2015; Shakil et al., 2016; Syed et al., 2017; Cheng et al., 2018). It makes sense that the patterns of FC with high similarity represent the same state and the connectivity patterns are the first pictures of the fluctuations of whole brain connection. However, high dimension in connection vectors may limit the findings, and the whole brain FC patterns are not well interpreted. What if using secondary measures of whole brain dynamic FC would yield meaningful representations of metastates? As is common knowledge, brain functional networks exhibit rich-club organization, whereby a small number of nodes tend to be connected densely. In fact, many studies have found that certain nodes or brain regions dynamically participate across different tasks (Schaefer et al., 2014; Bola and Sabel, 2015; Preti et al., 2017) or across different provincial communities (Hansen et al., 2015; Chen et al., 2017; Gordon et al., 2018). These indicated the potential feature of dynamic roles of nodes even in resting state. On the other hand, the node centrality is the secondary measure and can represent the topologic aspects of brain connectivity patterns. The regional activities or the regional signals are the origins of brain connectivity and the node centrality represents the significance of regional activities. Therefore, the patterns of regions/nodes would be reasonably more representative than the patterns of connectivity.

Overall, this paper aims to propose a method to extract the brain metastates using node centrality-based k-means clustering in resting state. Specifically, the node centrality scores were calculated as the degree-based eigenvector centrality (Correa et al., 2012; Meghanathan, 2015a, b) for each windowed FC matrix yielding a dynamic node centrality sequence. The metastates would be defined by the cluster centers after *k*-means. We expected that the metastates detected by the proposed method can represent meaningful information of brain function or physiological activities in resting state. Because of the lack of mathematical proof of metastates, experimental reliability analysis needs to be verified. Recently, there was a test-retest reliability study (Chao et al., 2018) about dynamic FC, providing the first insight into the reproducibility of dynamic FC but only focusing on the FC not the metastates. Therefore, a test-retest reliability experiment was performed to examine the repeatability of metastates. Furthermore, we further compared the hub distribution between functional metastates and the structural network to explore the potential relationship between them. Through this, we hopefully can verify the reliability of metastates extracted with the proposed method and delineate the potential mechanism of the functional dynamics in resting state.

## Materials and Methods

### Participants and MRI Acquisition

All recruited participants underwent rigorous clinical examinations and psychological evaluations and signed informed written consent. In total, 23 healthy adults (mean age: 23.6 years; range from 21 to 26 years; 12 female), without history of neurological or psychiatric disorders, with current physical and mental health and also with healthy living habits (no drugs, no alcohol addiction, no smoking, normal work and rest, and emotional stability) were included in this study. One week before MRI scanning, participants were told to keep normal emotion, sleeping and food intake (not too heavy, e.g., too hot or too salty). The study was approved by the medical ethics committee for research in humans of Tianjin First Central Hospital.

Magnetic resonance imaging images were acquired on a 3.0T Siemens scanner (Tim Trio, Germany) with a 32-channel head coil at Tianjin First Central Hospital. For each subject, there was a test-retest experiment: scanning twice with 1-week (7 days) interval at the same imaging site and same time (6:00 pm–9:00 pm) of day. Acquisitions included resting-state fMRI with echo-planar imaging (EPI) sequence, high-angular diffusion tensor imaging (DTI) with spin echo-echo planar imaging (SS-SE-EPI) sequence and anatomical T1 images with high-resolution 3-dimensional (3D) magnetization-prepared rapid acquisition with gradient echo (MPRAGE) sequence. Scanning settings for rs-fMRI were as follows: repetition time (TR) = 2.5 s, echo time (TE) = 30 ms, voxel size = 3.0 mm × 3.0 mm × 3.0 mm, flip angle (FA) = 80°, field of view (FOV) = 192 mm × 192 mm, matrix size = 64 × 64, number of slices = 28, slice thickness = 3 mm without interslice gap, scan time = 650 s, timepoints = 260. During scanning, participants were instructed to relax, keep their eyes open, try to keep their head and body still and not think anything special. Scanning settings for high-angular DTI were as follows: TR = 4000 ms, TE = 30 ms, number of slices = 45, slice thickness = 2 mm without interslice gap, voxel size = 2.0 mm × 2.0 mm × 2.0 mm; three unweighted b0 scans and 64 weighted diffusion scans with a weighting of 1000 s/mm^2^ were acquired within 12 min. Multiband acceleration sequencing was used with accelerated factor = 4. Scanning parameters for anatomical T1 images were acquired for anatomical reference and definition of the different structural nodes of the network, using the following scanning parameters: TR/TE = 10/4.6 ms, FOV = 240 mm × 240 mm, 176 slices covering the whole brain, 1.0 mm isotropic voxel size, about 5 min.

### Image Preprocessing

The fMRI data were preprocessed using the DPABI (V3.0) package^1^ (Kevin et al., 2009). Preprocessing steps included removing the first 10 volumes, slice-timing correction, head motion correction, linear trend removal, band-pass filtering with frequency of 0.02–0.1 Hz which depended on the size of sliding window (Hindriks et al., 2016), and spatial smoothing (FWHM = 6 mm full-width at half-maximum Gaussian kernel). Nuisance signals including mean signals from ventricles (CSF), white matter (WM), whole brain (global mean signal) and the 24 motion parameters (six motion parameters, derivative and the quadratic terms) were regressed out (Fox et al., 2005). There has long been controversy regarding global mean signal processing (Kevin et al., 2009 and Fox et al., 2009). Because global mean signal removal brings negative FCs, the global signal contains much non-neural information and is sensitive to head motion. However, researchers from the two perspectives come to a consensus (Murphy and Fox, 2016) about this issue: whether it is essential to do global signal removing really depends on the specific question. In current research, node-degree-based measures were obtained to capture the dynamic networks. It is commonly known that the global mean signal removing can greatly increase the specificity of the fMRI signal. Global mean signal removing will be beneficial for our purpose. In addition, removing the global mean signal can also reduce the impact associated with head motion. To further control head motion effects, we removed the volumes with frame-wise displacement (FD) higher than 0.3 mm and removed the subject remaining with less than 200 volumes. No significant differences were found in terms of mean FD (*p* = 0.811) or the number of censored volumes (*p* = 0.723) across all subjects between two scans by using paired *t*-test.

Diffusion tensor imaging images were preprocessed using DTI-Explore package^2^ (Leemans et al., 2009). Preprocessing steps included susceptibility distortions correction (estimating a field distortion map based on the three b0 images), eddy-current distortions and motion corrections (Andersson and Skare, 2002), a robust tensor fitting (Chang et al., 2005) and WM tract reconstruction based on the FACT (fiber assignment by continuous tracking) algorithm (Mori and Van Zijl, 2002; Mori et al., 2010). This procedure resulted in a large sample of all possible (reconstructable) fiber tracts of the brain network. A fiber streamline was stopped when the fiber track reached a voxel with a FA value 0.1 (indicating a low level of preferred diffusion within that particular voxel), when the trajectory of the traced fiber left the brain mask or when the fiber tract made a sharp turn of 45°.

For each subject, T1 images from two sessions were aligned and averaged for better quality. We utilized a two-step non-linear spatial registration method to transform the native functional or diffusional images to MNI space: firstly, native functional image (the first volume) or diffusional image (b0 image) was individually affined to the averaged T1 image; second, this natively averaged T1 image was nonlinearly registered based on the MNI-152 T1 template in FMRIB Software Library (FSL)^3^ software package (Linux, United Kingdom). Combining these two steps, we can easily transform all the well preprocessed functional images and diffusional measures into standard MNI space.

### Functional Network Construction

In this study, automated atlas labeling (AAL 90) (Tzourio-Mazoyer et al., 2002) was adopted to define the regions of functional networks. Each brain region in the AAL template was used as a regional mask to extract the time signal of BOLD functional data. We excluded the regions from the cerebellum to focus more attention on the brain patterns. Ninety columns of time signals were extracted and a 90 × 90 correlation matrix was calculated using Pearson correlation. Then FC matrixes were obtained by fisher z-transformation. The dynamic sequences of FC matrixes were obtained by the sliding window correlation method. There is still a lack of knowledge regarding what the best window length is and how it influences the results. A large number of previous studies (Hindriks et al., 2016; Chen et al., 2017, 2018) have converged to a short range from 50 to 60 s. Arbitrarily and also empirically, we fixed the length of the rectangle window as 60 s (width = 24 × TR), and the window was shifted with a step of 1 TR = 2.5 s (Hutchison et al., 2013a; Leonardi et al., 2013; Allen et al., 2014). Therefore, for one scan of each subject, a sequence of 232 FC matrixes were obtained.

The meaningless connections in static FC were removed to make the FC matrix to be spared or less redundant. Proportional thresholding on the weighted FC matrix was conducted based on the connection density, which is one of the two thresholding techniques of FC matrixes (the other is deterministic thresholding based on a FC strength). In order to select a proper density thresholding value, one sample *t*-test was done on the static FC matrixes to find the significant connections which were significantly >0 (FDR *q* < 0.05; focused on positive connections), and two binarized matrixes for each group were presented. A density thresholding value was selected referring to the densities of these group binarized matrixes, which were 0.38 and 0.41. In this paper, 40% of the connections—which had the higher FC strength—were retained (namely, 40% of the connections had higher FC strength and were set to one while the other 60% were set to zeros), yielding binarized connectivity matrixes. Density-based binarization can provide binarized FC matrixes which have the same number of connections. After the density-based thresholding, the FC weights were given back to the remaining connections, yielding weighted matrixes.

### Clustering Analysis

The degree-based measure of node centrality is a direct and local topologic measurement including degree centrality and eigenvector centrality. Degree centrality:

(1) $$C_{D}\,\left(i\right)=\sum_{i\neq j}^{\,}A_{i\,j}$$

Eigenvector centrality:

(2) $$C_{E}\,\left(i\right)=\frac{1}{\lambda_{1}}\,\sum_{j=1}^{N}A_{i\,j}\,x_{j}$$

Here, *Ax*_*j*_ = λ*x*_*j*_. “*A*” represents the FC matrix, “*x*_*j*_” and “λ,” respectively, represent the nonzero eigenvector and eigenvalue of “*A*,” “*i*” and “*j*” respectively, represent different nodes. Because degree centrality is too local, ignoring the importance of the nodes that the target node connects with, eigenvector centrality is considered here. These node centrality scores were calculated for each spared, weighted and windowed FC matrix, yielding a series of node centrality scores. The yielded node centrality time series represented the node centrality distribution of FC patterns at each windowed time. Then, every node centrality vector was normalized into a standard normal distribution *N*(0,1). We also compared the difference of this dynamic node centrality with different window length: 20, 30, 50, and 60 s (Supplementary Figure S1). The node centrality time series with all kinds of window lengths showed temporally repeated patterns. However, the periods of patterns were shorter, with 20 and 30 s, than that of other two window lengths, which were within the popularly used window length range. The 50 and 60 s patterns were quite similar.

**FIGURE 1:**
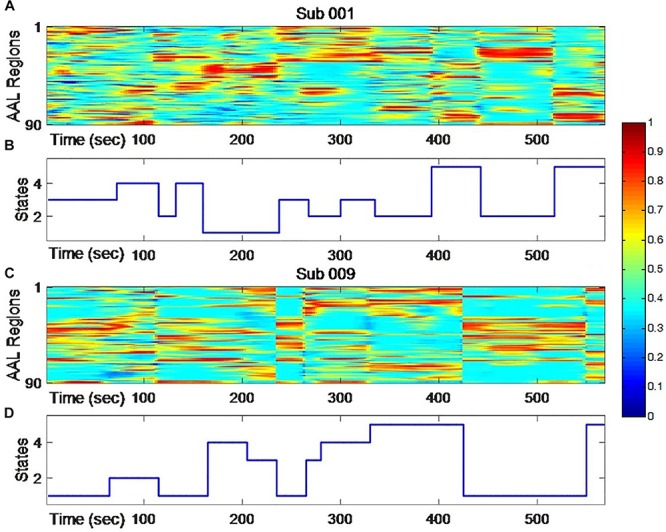
The dynamic changing of node centrality **(A,C)** and changing states of clustering analysis **(B,D)** for two typical subjects. The color represents normalized node centrality score and red means higher centrality.

Each node centrality vector of one windowed FC matrix was treated as one sample in clustering analysis. Datasets from scan I and scan II were treated as two independent groups to conduct the clustering analysis separately. Based on the k-means++ algorithm (Arthur and Vassilvitskii, 2007), the clustering results based on all vectors within one group were obtained first with randomized initialization (group-level clustering). And then the resulted cluster centers were used as the initial starts for a second round of clustering within each subject’s node centrality vectors in that group (individual clustering). K-means++ was reported to be more independent from the initial points than the original k-means clustering. Within the group-level clustering, an optimization about the number of clusters was conducted with elbow criterion based on the cluster validity index (Supplementary Figure S2). Finally, *k* = 5 was outperformed. For the distance measure in k-means, we tried several ones, and arbitrarily selected the correlation distance (1 – Pearson correlation) because of the better clustering and higher stability of the optimal number of clusters. Typical individual results were shown in Figure 1. The whole clustering strategies were done for each group separately. Different clusters or centers indicated that the extracted metastates and all the node centrality vectors recognized as the same cluster were averaged to represent the node centrality pattern of that metastate.

When the clustering was done, dwell time and transition time were calculated, which are typically and popularly used features to describe the dynamic of metastates (Allen et al., 2014; Chen et al., 2019). Dwell time was the total time that one metastate appears during the scan period (Supplementary Figure S3), which was calculated by the number of windows belonged to one cluster, or the number multiplied by TR (Damaraju et al., 2014; Mennigen et al., 2018; Xia et al., 2019). Transition time represented the times of transitions from one metastate to another during the scan period (Chen et al., 2019; Lee et al., 2019; Xia et al., 2019).

### Test-Retest Reliability Analysis

The intra-class correlation coefficient (ICC) (Bartko, 1966) is one of the reliability coefficient indexes to measure test-retest reliability. Bartko (1966) first used it to evaluate the reliability in 1966. Xi-Nian et al. (2010) and Zuo and Xing (2014) used ICC to analyze the test-retest reliability of various fMRI processing methods and indicators, which had important guiding significance for fMRI studies. ICC is equal to the individual variability divided by the total variability, and the value is between 0 and 1. A value of 0 represents completely untrusted, and 1 represents completely trusted. It is generally acknowledged that ICC < 0.4 indicates poor reliability and >0.75 indicates good reliability. ICC is defined as:

(3) $$\text{ICC}=\frac{\sum_{i=1}^{n}\left(x_{1\,i}-\bar{x}\right)\,\left(x_{2\,i}-\bar{x}\right)}{\left(n-1\right)\,s_{x}^{2}}$$

Here, *n* represents the total number of subjects; *x_1__*i*_* represents the first measurement of the *i*^*th*^ subject; *x_2__*i*_* represents the second measurement of the *i*^*th*^ subject; and $\bar{x}$ and *s*_*x*_ represent the mean value and the standard deviation of all observations, respectively. Before the ICC analysis, the Bartlett and Kolmogorov–Smirnov tests were applied to verify the heteroscedasticity and the normality of the data.

### Structural Network Construction

Based on DTI image analysis and fiber tracking, the direct structural connections were calculated. With the AAL-90 parcellation, 90 brain typical regions within individual native space were assigned and used to generate the structural connectivity according to the number of tracts between each pair of regions. Data processing was performed based on the whole brain fiber tracts using TrackVis software^4^.

### Regional Hub Nodes Analysis

A series of highly connected nodes, having high node degrees or high centralities, are identified as “brain hubs.” In this paper, we utilized a typical way to highlight the hub nodes. All nodes were ranked according to node centrality scores, and those higher than the mean up to one standard deviation were recognized as hub nodes (top-ranking nodes based on one standard deviation criteria) (Van Den Heuvel and Sporns, 2013; Dai et al., 2014; Oldham and Fornito, 2018). The node centrality scores representing each metastate were used to define the hub nodes of that metastate. Since the node centrality used here is a kind of degree-based centrality, these hub nodes mainly indicated the provincial hub characteristics. The hub nodes distribution of five metastates were extracted and presented in a 3D view. Also, the transition characteristics between different metastates were analyzed.

For the structural network, rich-club analysis (Heuvel Van Den and Olaf, 2011; Sharaev et al., 2018) was applied to delineate the highly connected sub-network known as rich-club, including all hubs. To define the rich-club, the steps included: (1) ranking nodes according to node centrality scores; (2) applying a threshold to define a subgraph that contains only more than a certain sorted node; (3) calculating the total weight of the connectivity between the reserved subgraph nodes; (4) calculating the weight sum of the same number of edges, which are the highest ranking weights in the complete network; and (5) calculating the ratio of steps 3 and 4. The rich-club coefficient is shown as follows:

(4) $$\emptyset^{w}\,\left(r\right)=\frac{W_{>r}}{\sum_{l=1}^{E_{>r}}w_{l}^{r\,a\,n\,k}},$$

where, *W*_*¿r*_ is the weight sum of the edges in the subgraph with nodal ranking higher than *r*, *E*_*¿r*_ is the number of these edges in the subgraph, and *w*^*rank*^ is one of the vectors whose weights are ranked from high to low. Due to random networks also showing an increasing function of $\emptyset_{\,\,\,}^{w}\,\left(r\right)$, $\emptyset_{\,\,\,}^{w}\,\left(r\right)$ is typically normalized by a set of comparable random networks of equal size and similar connectivity distribution, resulting in a normalized rich-club coefficient $\emptyset_{n\,o\,r\,m}^{w}\,\left(r\right)$, which was computed as:

(5) $$\emptyset_{n\,o\,r\,m}^{w}\,\left(r\right)=\frac{\emptyset^{w}\,\left(r\right)}{\emptyset_{r\,a\,n\,d}^{w}\,\left(r\right)},$$

Where, $\emptyset_{r\,a\,n\,d}^{w}\,\left(r\right)$ *w* rand is computed as the averaged rich-club coefficient from 1000 random networks preserving the same degree. This normalized rich-club coefficient gives a better indicator of the significance of the rich-club effect. For this metric, if for certain values of *r* then we have $\emptyset_{n\,o\,r\,m}^{w}\,\left(r\right)>1$, which denotes the presence of the rich-club effect.

## Results

### Clustering Results

**FIGURE 2:**
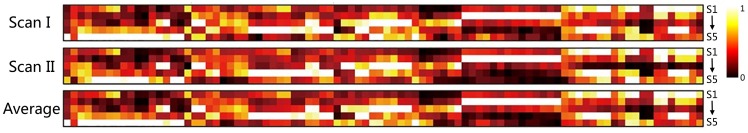
The clustering results for scan I, scan II and the averaged centers from two scans. The hotter regions represent a higher level of node centrality.

**FIGURE 3:**
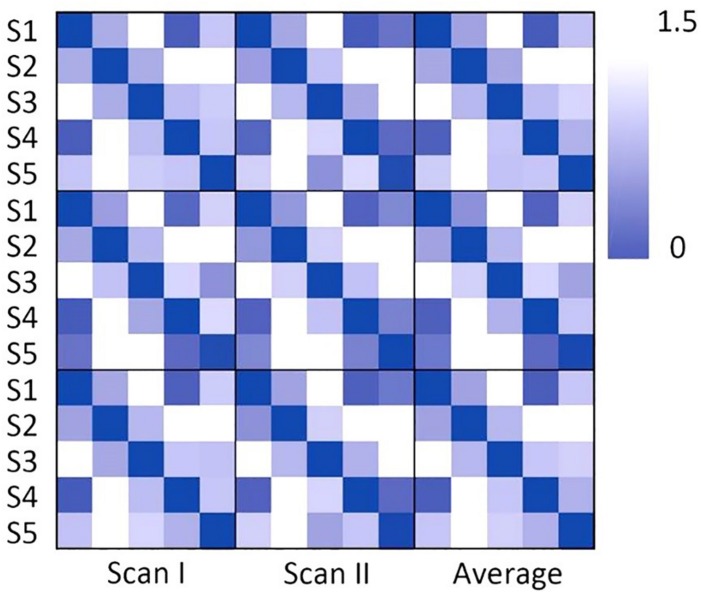
The correlation distance of the cluster center for scan I, scan II and the averaged centers from two scans. The dark color indicates the close correlation distance and high correlation, which represents that the two states can be considered to be the same state.

As shown in Figure 1, individual node centrality vectors and the corresponding time series of clusters’ labels from two typical subjects were presented. It could be found that the clustering results were well in accordance with the temporal changes of node centrality distributions. For two scans and the average group, the cluster centers were shown in Figure 2, including state 1 (S1), state 2 (S2), state 3 (S3), state 4 (S4), and state 5 (S5). The Figure 3 indicated distances between each of the two cluster centers. The dark color indicated low distance, which represented that the two clusters were closed metastates. On the contrary, the two states were quite different, which should be considered as two different metastates. There was high consistency between the clustering results from two scans, and the cluster centers from one group could exactly correspond to the similar one from the other group.

### Test-Retest Results

**FIGURE 4:**
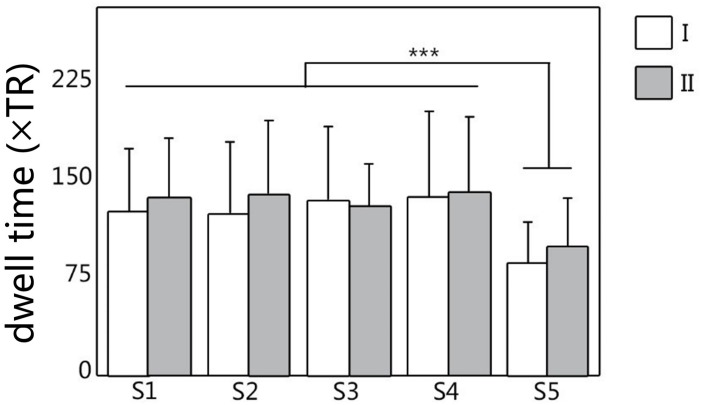
The dwell time of metastates under clustering, for all five metastates of two scans. White represents the dwell time of scan I, and gray represents the dwell time of scan II. ^∗∗∗^Represents significant difference.

**FIGURE 5:**
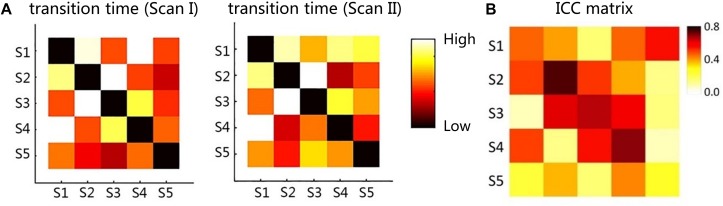
**(A)** The transition time matrix between different metastates for two scans. **(B)** The ICC matrix of two features (diagonal for dwell time and non-diagonal for transition time). Red to yellow indicates the time from high to low.

The dwell time and transition time were illustrated in Figures 4, 5A. In Figure 4, there was no significant difference between two scans in these two features, under FDR corrected *p* < 0.05. However, for both scans, after one-way ANOVA and *t*-test analysis, the dwell time of S5 was significantly lower than that of the other four (*p* < 0.001). The transition time matrixes, depicted in Figure 5A, also showed similar patterns between two scans. In the transition time matrix, the columns of a state represented that the time switched from other states to that state, and the rows of a state represented that the time moved from that state to other states. The ICC matrix (Figure 5B) of these two features (diagonal for dwell time and non-diagonal for transition time) revealed the reliability of the appearances of the observed metastates across 1-week interval scans. Results showed that there was high degree of reliability (ICC > 0.4) for dwell time of states 1–4 and most transition time between states. S5 showed relatively low reliability in its dwell time and high reliability only in transition from S1 to S5.

### Hub Nodes of Networks

**FIGURE 6:**
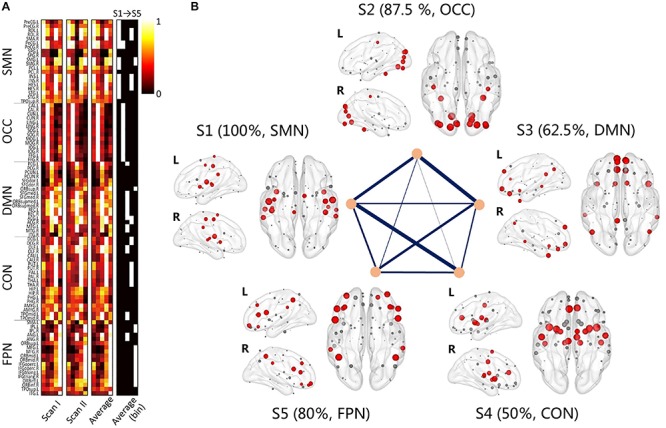
The group averaging results of the clustering. **(A)** The node centrality distribution for different groups, for Scan I, Scan II and the average separately; the binary one represents the hub regions for each metastate; **(B)** the 3D view of the hub regions for each metastate, red nodes represent the hub nodes; gray nodes represent the non-hub nodes. Transitions between different metastates are connected by straight lines, and thicker line represent the higher transition time. Each metastate shows the corresponding brain sub-networks and overlapping rate of hub regions with brain sub-networks.

**FIGURE 7:**
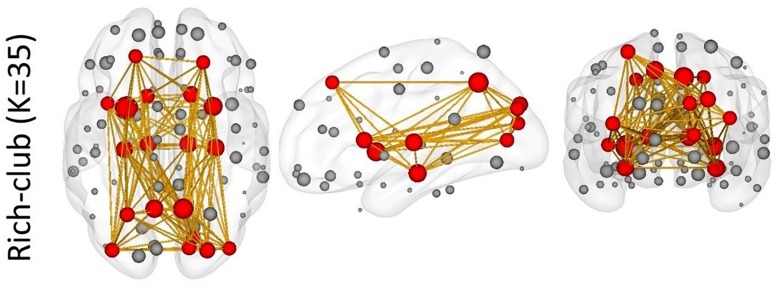
The rich-club results of the structural network. Red nodes represent the hub nodes of the structural network; gray nodes represent the non-hub nodes. The yellow lines depict the connectivity.

The node centrality distributions for different groups were showed in Figure 6A, for Scan I, Scan II and the average separately. Thresholding (mean + 1 standard deviation) the hub nodes on the averaged node centrality scores provided the binarized map indicating the hub nodes for each metastate, as well as the corresponding spatial visualization with glass brain in Figure 6B. The Figure 6B showed the hub node distribution of each metastate, including S1, S2, S3, S4, and S5. Moreover, the transition characteristics between different metastates (the thicker line represented the higher transition time) were described. Also, the overlapping rates of the hub regions with brain intrinsic sub-networks were shown. The detailed information of these hub nodes for each metastate is listed in Table 1 along with one of the previously well-established brain intrinsic sub-networks: the frontoparietal network (FPN), occipital network (OCC), sensorimotor network (SMN), default mode network (DMN) and cingulo-opercular network (CON, mainly includes the subcortical nucleus). Segmented based on AAL, these hub patterns of each metastate could be uniquely assigned as one of these intrinsic networks, according to the overlapping rate between hub nodes and intrinsic sub-network regions. In Table 1, the underlined regions are the most overlapping regions between hub nodes and intrinsic network (state 1: 17/17 with SMN; state 2: 14/16 with OCC; state 3: 10/16 with DMN; state 4: 9/18 with CON; state 5: 12/15 with FPN). The hub nodes of the structural network are also listed in Table 1 and visualized in Figure 7, which resulted from rich-club analysis. The hub regions shared with the structural network for each metastate are boldfaced in Table 1.

**TABLE 1:** Hub regions of both metastate and structural network.

**Network**	**Hub Regions**
State 1 (SMN)	PreCG.L, PreCG.R, ROL.L, ROL.R, **SMA.L**, **SMA.R**, INS.L, INS.R, PoCG.L, PoCG.R, SMG.L, SMG.R, PCL.L, HES.L, HES.R, STG.L, STG.R
State 2 (OCC)	CAL.L, CAL.R, CUN.L, CUN.R, LING.L, LING.R, **SOG.L**, **SOG.R**, MOG.L, MOG.R, IOG.L, IOG.R, FFG.L, FFG.R, PoCG.L, PoCG.R
State 3 (DMN)	PCG.L, **SFGmed.L**, **SFGmed.R**, ORBsupmed.L, ORBsupmed.R, REC.L, REC.R, ACG.L, MTG.L, MTG.R, OLF.L, OLF.R, TPOmid.L, TPOmid.R, ANG.L, ANG.R
State 4 (CON)	ROL.L, ROL.R, **SMA.R**, INS.L, INS.R, HES.L, HES.R, ACG.L, ACG.R, DCG.L, **DCG.R**, _CAU.L, PUT.L, PUT.R, PAL.L, PAL.R, THA.L, THA.R
State 5 (FPN)	SMG.L, SMG.R, ORBsup.R, IPL.L, IPL.R, MFG.L, MFG.R, ORBmid.L, ORBmid.R, FGoperc. L, IIFGoperc.R, IFGtriang. L, IFGtriang.R, ORBinf.L, ORBinf.R
Structural	SFGdor.L, SFGdor.R, **SMA.L**, **SMA.R, SFGmed.L**, **DCG.R**, **SOG.L**, **SOG.R**, MOG.L, PCUN.L, PCUN.R, **PUT.L**, **PUT.R**, **THA.L**, **THA.R**,

## Discussion

In this paper, we proposed a method to extract metastates based on the node centrality of the dynamic functional networks and assessed the appearance of these metastates with test-retest across a 1-week interval. To our knowledge, this is the first study to analyze the repeatability of metastates of dynamic functional networks with time interval in the resting state. Furthermore, we also found the coupling relationship between dynamic functional networks and the structural network at the hub regions level. Several main findings are as follows: (1) the proposed method showed high reliability in individual metastates extracted across a 1-week interval; (2) the hub regions of each metastate highly overlapped with the intrinsic functional brain subnetworks; (3) the hubs of metastates were highly overlapped with the structural core network. It can be speculated that the dynamic transitions between metastates are potentially associated with the core structure of the structural network, indicating structural constraint.

### Repeatability of Proposed Method

Previous studies suggested that a high frequency of FC state transitions existed in the brain (Damaraju et al., 2014; Li et al., 2017; Marusak et al., 2017), but the stability of these states as well as their transitions have not been proved. In our study, the changes of dwell time and transition time can reflect the temporal characteristics of functional metastates, which indicates that metastates show a process of stable dynamic changes over time. As shown in Figures 4, 5, according to the metastate results of the same group of subjects at two scans in different time, the repeatability of the metastate time series can be detected. Dwell time can demonstrate the importance of a certain state in the temporal series of brain dynamic function. Longer dwell time indicates that the brain function corresponding to this state is more dominant. Respectively, for five metastates, there is no statistical significance in dwell time between scan I and scan II. This result indicates the stability of the metastate. At the same time, S5 is significantly different from the other states, which demonstrates that S5 is not active at resting state (Figure 4). The transition time indicates the information exchange and cooperation mechanism between metastates (Chen et al., 2019). In the brain dynamic functional network time series during resting state, the more frequently the state is transiting indicates that there is more information exchange in this state, which may involve an internally close collaboration or interaction mechanism. It is observed that the average number of metastate transitions is highly consistent (Figure 5A). Moreover, a majority of ICCs are higher than 0.4, which illustrates good reliability (Figure 5B). Interestingly, compared with other states, the transitions of S5 shows a low reliability, which may be related to the instability of S5. It is concluded that the dynamic transition rules of the metastates obtained by the two scans are almost the same, which indicates that the time series of brain metastate transitions in the same individual have good repeatability in different time periods.

### Specific Representations of Functional Metastates

The hub nodes of each FC network metastate show the particularity in spatial distribution. Considering the hub node distribution of each metastate and functional subnetwork, it is found that the hub node distribution is highly consistent with the functional subnetwork nodes. Hub nodes for each metastate are mainly located in the corresponding subnetwork. Specifically, five metastates correspond to specific networks with FC, such as the FPN (Emerson and Cantlon, 2012; Di and Biswal, 2015; Lee and Telzer, 2016), OCC (Yan et al., 2011; Damoiseaux et al., 2016; Chen et al., 2017; Zhang et al., 2017), SMN (Jolles et al., 2011; Pujol et al., 2014; He et al., 2016; Syed et al., 2017), DMN (Betzel et al., 2014; Wu et al., 2014; Jiao et al., 2016, 2017) and (CON, mainly includes the subcortical nucleus) (Betzel et al., 2014; Luca et al., 2014; Raichlen et al., 2016; Long et al., 2017), respectively (Figure 6). Accordingly, the dynamic changes of the brain functional network reflect the characteristics whereby different functional modules are “activated” alternately in a certain time series.

It is found that the hub node distributions of five metastates respectively, correspond with five intrinsic functional networks, which reveals the physiological significance of metastates. Among them, S5 corresponds to the FPN, which has a low repeatability. As the main network in higher the cognitive and thinking consciousness processes, the FPN is susceptible to brain activity at resting state (Zanto and Adam, 2013; Gulbinaite et al., 2014; Alarcón et al., 2018). A low repeatability of S5 can be explained in that the resting state is an ambiguous and imprecise state of brain function. Furthermore, S5 is relatively less active in resting state with a lower dwell time, which further indicates that the FPN is a brain network involved in higher cognitive processes. The changes of dynamic network time series seem to imply that the brain shows dynamic “activation” characteristics of different intrinsic networks or cognitive resources. On the other hand, the intrinsic networks or network modularization structure are closely related to the rotational activity of the dynamic local brain regions. Therefore, it is speculated that compared with the traditional network modularization organization of static FC, the single spatial integration and separation characteristics reveal the internal mechanism. This particular pattern of temporal and spatial organization may better reflect the organization and operation mechanism of brain networks. It is interesting to find that the hub nodes of metastate are not fully consistent with the intrinsic network of brain regions. Further analyzing the distribution of hub nodes, we can find that the hub nodes of each metastate respectively, belong to local hubs and global hubs. The local hub is responsible for information integration within the network, and the global hub is responsible for information integration between networks. Thus, we can speculate that the changes of hub nodes reflect the integration ability of local brain functional resources, and it is simultaneously constrained by the global network structure.

### Coupling With the Structural Network

Through the rich-club analysis, we extracted the structural core of the structural network (Figure 7). The most important nodes were obtained in the structural network, which are highly overlapped with hub nodes. In addition, a small number of hub nodes in each metastate belonged to the rich-club. It is indicated that the dynamic changes of functional metastates are the spontaneous transition of the intrinsic function resources, which are captured based on the node centrality. This transition mechanism relies on the structural core of the structural network, which plays an important connector role in the metastate transition process (Hagmann et al., 2008; Yong et al., 2009; Heuvel Van Den and Olaf, 2011). At the same time, the constant changes of metastates reflect the feeder effect of local intrinsic networks. The connect-feed theory simplifies brain network connectivity from an information-processing perspective (Bullmore and Sporns, 2009). Connective core nodes and hub nodes are defined as the connectors, which have the effect of globally connecting different modules. Connectivity that connects edge nodes or local network nodes to core nodes or hub nodes is known as feeder, which can transfer local information to advanced network structure.

## Limitations

We have designed a test-retest study to assess the reliability of functional metastates, but there are still several aspects that need further improvements. First, strictly inclusive participants are of a relatively small size. Although we have some potential influential factors that may affect the results, it is still hard to be sure because of the small sample size. Specially, it is more focused on the age at about 20 s and may not be represented. To further corroborate our results and elucidate the spontaneous fluctuations of the FC through metastates transitions, a better study should be performed to follow subjects from wide ages. A study a with large sample will also improve estimates of FC variability and permit patterns of connectivity, which may be critical for future investigations. Second, as mentioned in the discussion section, the brain parcellation atlas used is a commonly used one, and there are many finer templates with higher spatial resolution and more detailed or specific divisions of brain regions. With finer parcellation, it is probable to obtain more spatially dependent patterns represented as metastates and provide more information about coupling between dynamic function in resting state and intrinsic structure. It is also another powerful way to verify the reliability of the proposed method at different spatial levels of brain parcellation. Third, how the hub regions are identified here is not rigorous, but relatively comparable. With that said, we used the normalized rich-club coefficient to find the hubs of the structural network, and spurious hubs nodes will probably be found. At the same time, there were many details that were arbitrary and tricky from the perspective of more rigorous thinking, for example the functional network thresholding, the sliding window size and the fMRI preprocessing. This makes it difficult to draw a strong conclusion. However, the methods here provide a general method and insight view of the dynamic hub nodes of functional networks. Future work should use stricter methods to identify the hub nodes, for example using non-parametrical testing and multiple comparison correction. Fourth, the exact sliding window size is important and should be carefully considered in dynamic FC research. In this work, we arbitrarily chose an empirically used window length in previous literatures without further exploring the potential effects for varied window lengths. Future work on the effect of window size on metastate extraction is needed. What is more important about this is whether the sliding window correlation method reflects the dynamic FC, which has been the focus of several studies (Leonardi and Van De Ville, 2015; Hindriks et al., 2016) and resulted in two sides. For example, with surrogate data, Hindriks et al. (2016) concluded that with the sliding window-based method it was hard to reflect the dynamic FC, and Leonardi suggested that an extra 0∼1/w low frequent pass filtering on the sequence of dynamic FC can reduce spurious information about dynamic FC, even though plenty of studies have found meaningful things about dynamic FC. In our thoughts, what really mattered is to know what kind of or how to describe the characteristics of dynamic FC in resting state. In any case, a surrogate can never represent real fMRI data and we also never know the exact information underlying our brain function. However, with careful thinking, we might still explore the nature of dynamic FC in practice.

## Conclusion

In conclusion, we proposed a clustering method to extract metastates based on the node centrality of the dynamic functional networks, assessed the dynamic features of these metastates in resting state across a 1-week interval and further explored the possible meanings of these metastates. These metastates were repeatable and highly related to the intrinsic subsystems of brain function in resting state. Considering the overlapping of the hub nodes between metastates and the structural network, we also speculated that dynamic functional network metastates were coupled with or constrained by the structural network. We might further conclude that the metastates, or possible sub-systems, interacted with each other in an alternate provincial centralization under the core frame of the structural network. In additional, node-based representation of dynamic function networks, as well as metastates, might provide a new useful insight into the underlying information of spontaneous dynamics in resting state networks.

## Ethics Statement

All subjects gave written informed consent in accordance with the Declaration of Helsinki. The protocol was approved by the medical ethics committee for research in humans of Tianjin First Central Hospital.

## Author Contributions

XZ and QW were in charge of the analysis work and wrote the manuscript. YC and QW processed all the image data and conducted the main analysis work. YC and HN collected the MRI data. XS and HN provided some useful guidance and ideas. YC, XZ, and DM designed and provided the original idea. HN, XZ, and DM sponsored the whole research.

## Conflict of Interest Statement

The authors declare that the research was conducted in the absence of any commercial or financial relationships that could be construed as a potential conflict of interest.

## Footnotes

1 http://rfmri.org/dpabi

2 http://www.exploredti.com

3 https://fsl.fmrib.ox.ac.uk/fsl/fslwiki/

4 http://trackvis.org/
