# Supplementary material for: Hub Patterns-Based Detection of Dynamic Functional Network Metastates in Resting State: A Test-Retest Analysis
Source: Front Neurosci. 2019 Sep 11;13:856. doi: 10.3389/fnins.2019.00856 (PMC6749078; doi:10.3389/fnins.2019.00856)
Supplement: Supplementary file 1 [file Data_Sheet_1.docx]

**Supplementary Figure 1**. The dynamic node centrality time series calculated with different sliding window length, 20 sec, 30 sec, 50 sec and 60 sec. The color represents normalized node centrality score and red means higher centrality.


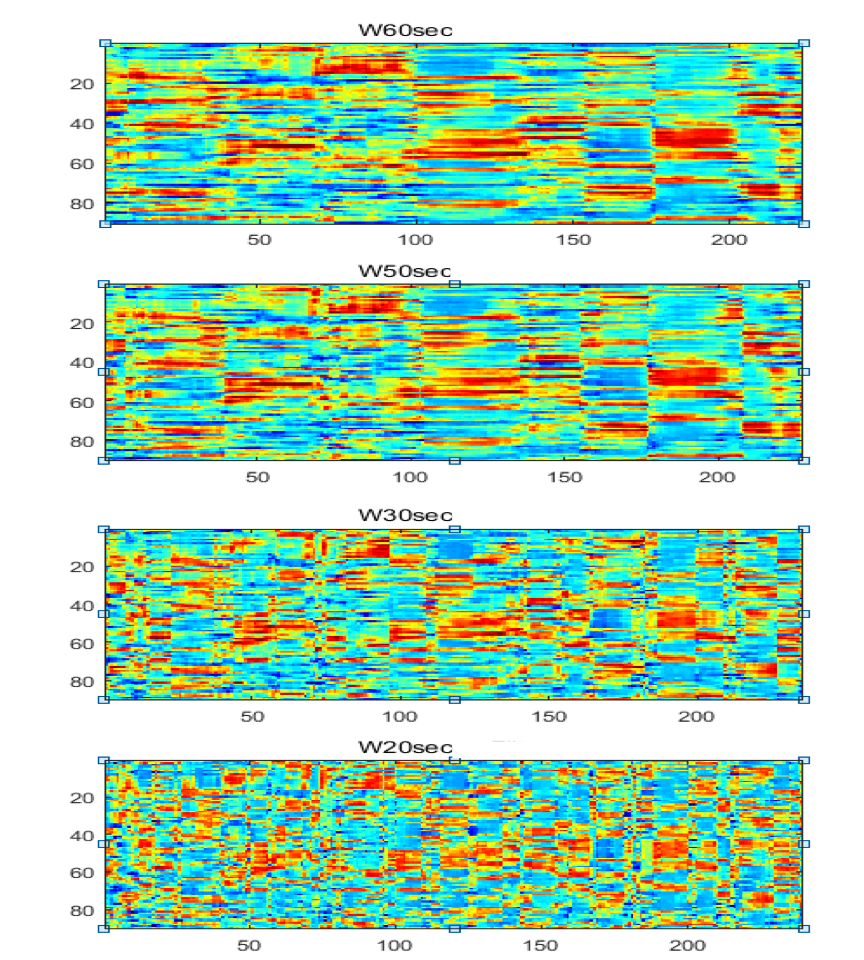


**Supplementary Figure 2**. The clustering number results of two scans. Red nodes are the optimal clustering number, and it can be understood that there are five most important microstates.


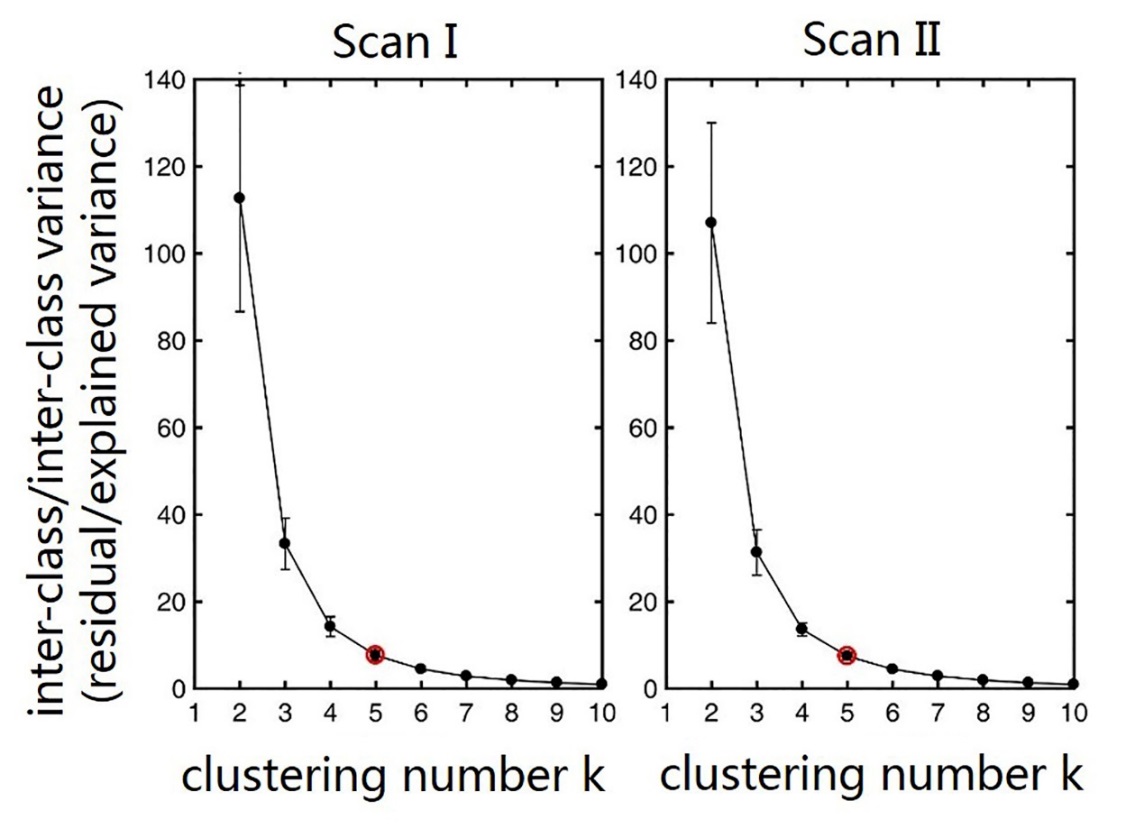


**Supplementary Figure 3**. Illustration of the dwelling time and transition time for five microstates. The red curve shows the transition of all states during the scanning time. The green curve represents the transition time from S5 to S1. The blue curves represent the dwelling time of S2 in throughout the transition process.

**
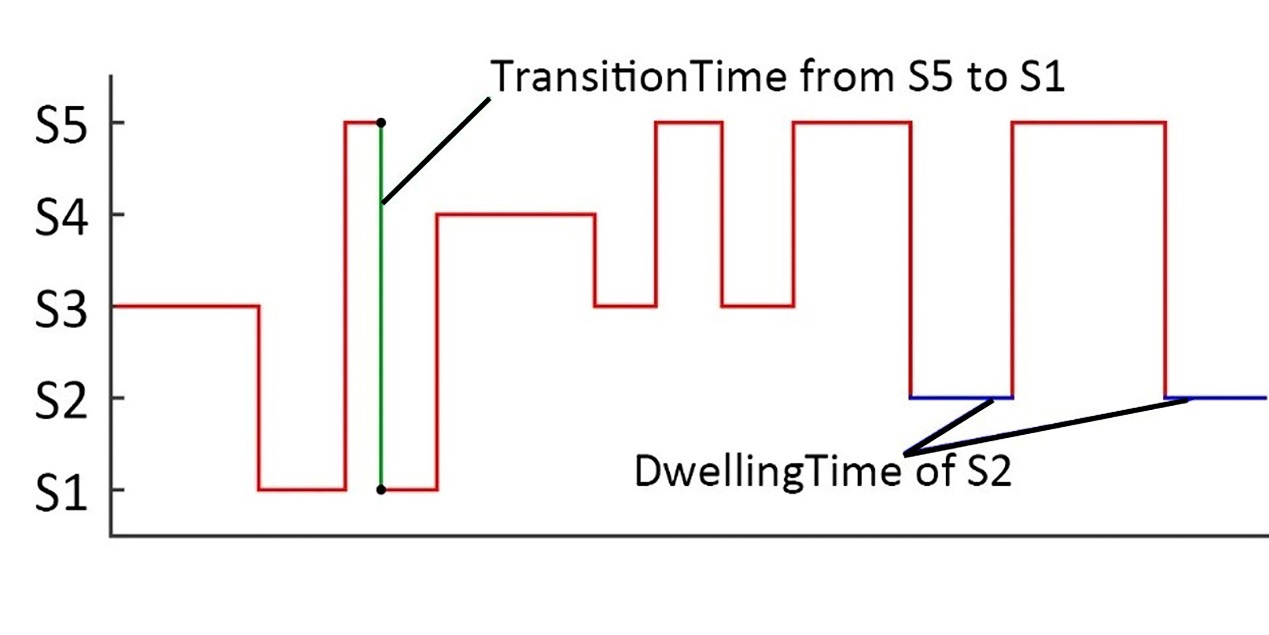
**
